# Ethyl 3-(3-oxo-3,4-dihydro­quinoxalin-2-yl)propano­ate

**DOI:** 10.1107/S1600536810047094

**Published:** 2010-11-20

**Authors:** Waqar Nasir, Munawar Ali Munawar, Ahmad Adnan, Saeed Ahmad, Mohammad Akbar

**Affiliations:** a Institute of Chemistry, University of Punjab, New Campus, Lahore, Pakistan; b Department of Chemistry, GC University, Lahore 54000, Pakistan; c Department of Chemistry, Gomal University, D. I. Khan, Pakistan

## Abstract

In the title compound, C_13_H_14_N_2_O_3_, the fused ring system is almost planar (r.m.s. deviation = 0.015 Å). The r.m.s. deviation for all the non-H atoms of the mol­ecule is 0.065Å. In the crystal, N—H⋯O and C—H⋯O hydrogen bonds generate polymeric chains along the *b* axis containing alternating centrsymmetric *R*_2_^2^(8) and *R*_2_^2^(20) loops.

## Related literature

For the synthesis, see: Taylor *et al.* (1965 ▶). For the biological activity of benzopyrazines, see: Sona *et al.* (1998 ▶); Cai *et al.* (1997 ▶); Toshima *et al.* (2003 ▶); Benbow *et al.* (2007 ▶); Sarges *et al.* (1990 ▶); Smits *et al.* (2008 ▶); Tandon *et al.* (2006 ▶).
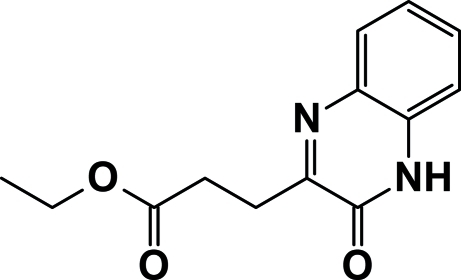


## Experimental

### 

#### Crystal data

C_13_H_14_N_2_O_3_
*M* *_r_* = 246.26
Monoclinic, 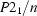

*a* = 8.3138 (6) Å
*b* = 13.6868 (8) Å
*c* = 10.8189 (8) Å
β = 102.002 (3)°
*V* = 1204.16 (14) Å^3^
*Z* = 4
Mo *K*α radiation
μ = 0.10 mm^−1^
*T* = 296 K
0.37 × 0.29 × 0.23 mm

#### Data collection

Bruker Kappa APEXII CCD diffractometer
Absorption correction: multi-scan (*SADABS*; Bruker, 2007 ▶) *T*_min_ = 0.965, *T*_max_ = 0.978
11123 measured reflections
2938 independent reflections
1370 reflections with *I* > 2σ(*I*)
*R*_int_ = 0.060

#### Refinement

*R*[*F* ^2^ > 2σ(*F* ^2^)] = 0.068
*wR*(*F* ^2^) = 0.216
*S* = 0.96
2938 reflections
167 parameters
H atoms treated by a mixture of independent and constrained refinement
Δρ_max_ = 0.44 e Å^−3^
Δρ_min_ = −0.30 e Å^−3^

### 

Data collection: *APEX2* (Bruker, 2007 ▶); cell refinement: *SAINT* (Bruker, 2007 ▶); data reduction: *SAINT*; program(s) used to solve structure: *SHELXS97* (Sheldrick, 2008 ▶); program(s) used to refine structure: *SHELXL97* (Sheldrick, 2008 ▶); molecular graphics: *ORTEP-3* (Farrugia, 1997 ▶) and *PLATON* (Spek, 2009 ▶); software used to prepare material for publication: *WinGX* (Farrugia, 1999 ▶) and *PLATON*.

## Supplementary Material

Crystal structure: contains datablocks I, global. DOI: 10.1107/S1600536810047094/hb5740sup1.cif

Structure factors: contains datablocks I. DOI: 10.1107/S1600536810047094/hb5740Isup2.hkl

Additional supplementary materials:  crystallographic information; 3D view; checkCIF report

## Figures and Tables

**Table 1:** Hydrogen-bond geometry (Å, °)

*D*—H⋯*A*	*D*—H	H⋯*A*	*D*⋯*A*	*D*—H⋯*A*
N1—H1*N*⋯O1^i^	0.96 (6)	1.87 (6)	2.827 (3)	179 (5)
C3—H3⋯O3^ii^	0.93	2.51	3.426 (4)	170

Symmetry codes: (i) 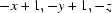; (ii) 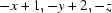.

**Fig. 1.**
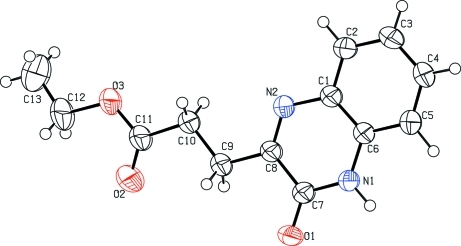
The structure of (I) with displacement ellipsoids drawn at the 50% probability level.

**Fig. 2.**
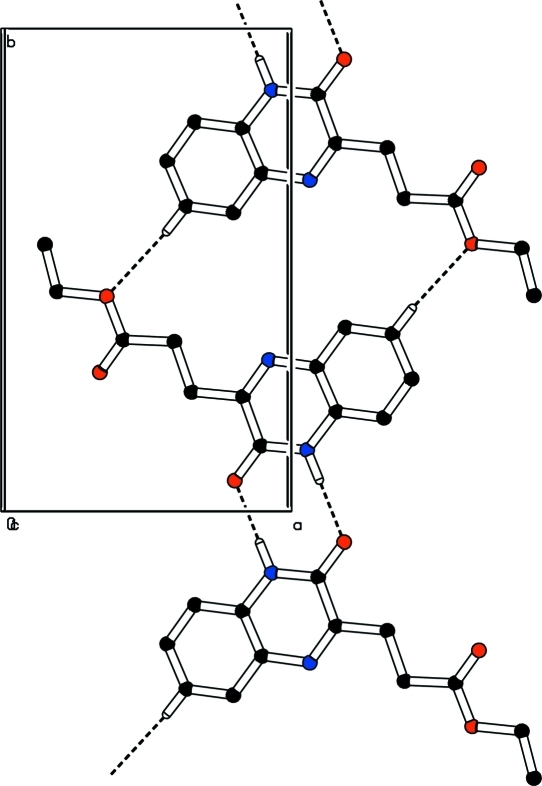
Unit cell packing diagram showing the hydrogen bonding using dashed lines, the hydrogen atoms not involved in hydrogen bonding have been omitted.

## supplementary crystallographic information

### Comment

Benzopyrazine constitute an important class of nitrogen containing heterocyclic compounds. Literature has shown that these compounds possess a vide variety of applications from pharmaceutical to agricultural fields. Several benzopyrazines have been reported as anti-bacterial (Sona *et al.*, 1998), anti-convulsant(Cai *et al.*, 1997), anti-cancer (Toshima *et al.*, 2003), antidiabetic(Benbow *et al.*, 2007), antidepressant(Sarges *et al.*, 1990), antifungal(Tandon *et al.*, 2006), anti-inflammatory (Smits *et al.*, 2008), etc. The present work is based on the synthesis of pyrazines derivatives which may possess enhanced pharmaceutical activities.

The title compound (I) is structurally looks like planer but the dihedral angle between the two fused rings i.e. aromatic ring (C1/C2/C3/C4/C5/C6) and pyrazine ring (C1/C6/N1/N2/C7/C8) is  1.46 (11)%. The planer ester moiety attached to the C8 is oriented at dihedral angle of 5.70 (14)% and 7.13 (13)% with respect to the aomatic and pyrazine rings respectively. The cyclic carboxamide functional group from the pyrazine rings forms dimers through N—H···O type hydrogen bonding interaction which further connects through weak C—H···O type hydrogen bonding interaction to form the polymeric chain along b axes (Fig. 2 Table 1).

### Experimental

To the suspension of 3-(3-oxo-3,4-dihydroquinoxalin-2-yl)propanoic acid (Taylor *et al.*, 1965) (5 g, 0.023 mol) in absolute ethanol (100 ml)  was added 3N H_2_SO_4_ (10 ml) and reaction mixture was refluxed for four hours. Solution was then concentrated under reduced pressure and neutralized with sodium bicarbonate solution to dissolve any unreacted acid. The precipitates were filtered under reduced pressure and washed with excess of water. The resulting ester was then recrystallized in absolute ethanol to yield colourless needles of (I). The product melted at 173-175 C (lit mp 160-162 C). (95% yield).

### Refinement

All the C—H H-atoms were positioned with idealized geometry with C—H = 0.93 Å , C—H = 0.96 Å and C—H = 0.97 Å and were refined using a riding model with *U*_iso_(H) = 1.2 *U*_eq_(C) for aromatic and methylene and *U*_iso_(H) = 1.5*U*_eq_(C)  for methyl C atoms. The N—H H atom were located in difference map with C—H = 0.96 Å with *U*_iso_(H) = 1.2 *U*_eq_(N). The reflection (011) was omitted during refinement as it was obscured by the beam stop.

### Crystal data

**Table:**

C_13_H_14_N_2_O_3_	*F*(000) = 520
*M**_r_* = 246.26	*D*_x_ = 1.358 Mg m^−^^3^
Monoclinic, *P*2_1_/*n*	Mo *K*α radiation, λ = 0.71073 Å
Hall symbol: -P 2yn	Cell parameters from 1697 reflections
*a* = 8.3138 (6) Å	θ = 2.8–24.6°
*b* = 13.6868 (8) Å	µ = 0.10 mm^−^^1^
*c* = 10.8189 (8) Å	*T* = 296 K
β = 102.002 (3)°	Cut needle, colourless
*V* = 1204.16 (14) Å^3^	0.37 × 0.29 × 0.23 mm
*Z* = 4	

### Data collection

**Table:**

Bruker Kappa APEXII CCD diffractometer	2938 independent reflections
Radiation source: fine-focus sealed tube	1370 reflections with *I* > 2σ(*I*)
graphite	*R*_int_ = 0.060
φ and ω scans	θ_max_ = 28.3°, θ_min_ = 3.5°
Absorption correction: multi-scan (*SADABS*; Bruker, 2007)	*h* = −11→11
*T*_min_ = 0.965, *T*_max_ = 0.978	*k* = −11→18
11123 measured reflections	*l* = −14→14

### Refinement

**Table:**

Refinement on *F*^2^	Primary atom site location: structure-invariant direct methods
Least-squares matrix: full	Secondary atom site location: difference Fourier map
*R*[*F*^2^ > 2σ(*F*^2^)] = 0.068	Hydrogen site location: inferred from neighbouring sites
*wR*(*F*^2^) = 0.216	H atoms treated by a mixture of independent and constrained refinement
*S* = 0.96	*w* = 1/[σ^2^(*F*_o_^2^) + (0.0982*P*)^2^ + 0.5331*P*] where *P* = (*F*_o_^2^ + 2*F*_c_^2^)/3
2938 reflections	(Δ/σ)_max_ < 0.001
167 parameters	Δρ_max_ = 0.44 e Å^−^^3^
0 restraints	Δρ_min_ = −0.30 e Å^−^^3^

### Special details

**Table:**

Geometry. All s.u.'s (except the s.u. in the dihedral angle between two l.s. planes) are estimated using the full covariance matrix. The cell s.u.'s are taken into account individually in the estimation of s.u.'s in distances, angles and torsion angles; correlations between s.u.'s in cell parameters are only used when they are defined by crystal symmetry. An approximate (isotropic) treatment of cell s.u.'s is used for estimating s.u.'s involving l.s. planes.
Refinement. Refinement of F^2^ against ALL reflections. The weighted R-factor wR and goodness of fit S are based on F^2^, conventional R-factors R are based on F, with F set to zero for negative F^2^. The threshold expression of F^2^ > 2σ(F^2^) is used only for calculating R-factors(gt) etc. and is not relevant to the choice of reflections for refinement. R-factors based on F^2^ are statistically about twice as large as those based on F, and R- factors based on ALL data will be even larger.

### Fractional atomic coordinates and isotropic or equivalent isotropic displacement parameters (Å^2^)

**Table:**

	*x*	*y*	*z*	*U*_iso_*/*U*_eq_	
N2	0.5708 (3)	0.81339 (16)	0.0220 (2)	0.0448 (6)	
C6	0.3369 (3)	0.70659 (19)	−0.0524 (3)	0.0427 (7)	
C1	0.4043 (3)	0.79969 (19)	−0.0334 (3)	0.0436 (7)	
C8	0.6615 (3)	0.73747 (19)	0.0532 (3)	0.0423 (7)	
C5	0.1719 (4)	0.6942 (2)	−0.1080 (3)	0.0534 (8)	
H5	0.1271	0.6318	−0.1199	0.064*	
N1	0.4382 (3)	0.62700 (17)	−0.0166 (2)	0.0478 (7)	
O3	1.1357 (2)	0.94521 (16)	0.1990 (3)	0.0763 (8)	
C7	0.5997 (3)	0.63615 (19)	0.0353 (3)	0.0448 (7)	
C2	0.3048 (3)	0.8807 (2)	−0.0717 (3)	0.0518 (8)	
H2	0.3487	0.9433	−0.0600	0.062*	
C9	0.8402 (3)	0.7474 (2)	0.1117 (3)	0.0485 (8)	
H9A	0.9034	0.7137	0.0589	0.058*	
H9B	0.8613	0.7151	0.1933	0.058*	
C10	0.9003 (3)	0.8516 (2)	0.1298 (3)	0.0503 (8)	
H10A	0.8863	0.8834	0.0481	0.060*	
H10B	0.8348	0.8868	0.1797	0.060*	
O1	0.6900 (2)	0.56381 (14)	0.0636 (2)	0.0593 (7)	
C11	1.0773 (4)	0.8557 (2)	0.1947 (3)	0.0563 (9)	
O2	1.1589 (3)	0.78799 (19)	0.2404 (3)	0.0934 (10)	
C3	0.1417 (4)	0.8677 (2)	−0.1268 (3)	0.0591 (9)	
H3	0.0754	0.9219	−0.1518	0.071*	
C12	1.3079 (4)	0.9554 (3)	0.2601 (4)	0.0911 (14)	
H12A	1.3271	0.9249	0.3428	0.109*	
H12B	1.3760	0.9226	0.2102	0.109*	
C4	0.0752 (4)	0.7746 (2)	−0.1455 (3)	0.0577 (9)	
H4	−0.0351	0.7667	−0.1834	0.069*	
C13	1.3500 (6)	1.0526 (4)	0.2726 (6)	0.131 (2)	
H13C	1.3410	1.0811	0.1904	0.196*	
H13A	1.4611	1.0587	0.3193	0.196*	
H13B	1.2773	1.0858	0.3169	0.196*	
H1N	0.394 (6)	0.562 (5)	−0.033 (5)	0.157*	

### Atomic displacement parameters (Å^2^)

**Table:**

	*U*^11^	*U*^22^	*U*^33^	*U*^12^	*U*^13^	*U*^23^
N2	0.0344 (13)	0.0382 (13)	0.0576 (16)	−0.0021 (10)	−0.0004 (11)	0.0004 (11)
C6	0.0340 (15)	0.0374 (15)	0.0542 (18)	0.0006 (12)	0.0034 (13)	0.0025 (13)
C1	0.0362 (16)	0.0384 (16)	0.0526 (18)	0.0021 (12)	0.0010 (13)	0.0010 (13)
C8	0.0357 (15)	0.0362 (15)	0.0507 (17)	0.0023 (11)	−0.0009 (13)	0.0001 (13)
C5	0.0374 (17)	0.0458 (18)	0.073 (2)	−0.0046 (13)	0.0014 (15)	−0.0021 (15)
N1	0.0332 (13)	0.0357 (13)	0.0680 (17)	−0.0008 (10)	−0.0049 (11)	0.0009 (11)
O3	0.0340 (12)	0.0536 (14)	0.127 (2)	−0.0050 (10)	−0.0155 (12)	−0.0091 (14)
C7	0.0396 (16)	0.0347 (15)	0.0564 (19)	−0.0002 (12)	0.0015 (14)	−0.0025 (13)
C2	0.0428 (18)	0.0371 (16)	0.071 (2)	0.0018 (12)	0.0008 (15)	0.0003 (14)
C9	0.0339 (16)	0.0423 (16)	0.065 (2)	0.0019 (12)	−0.0004 (14)	−0.0012 (14)
C10	0.0336 (16)	0.0424 (17)	0.069 (2)	0.0006 (12)	−0.0035 (14)	0.0018 (15)
O1	0.0428 (12)	0.0353 (11)	0.0905 (17)	0.0076 (9)	−0.0075 (11)	−0.0010 (10)
C11	0.0356 (16)	0.052 (2)	0.074 (2)	0.0011 (14)	−0.0057 (15)	−0.0044 (16)
O2	0.0467 (15)	0.0687 (17)	0.143 (3)	0.0075 (12)	−0.0295 (15)	0.0194 (16)
C3	0.0420 (18)	0.0458 (18)	0.083 (2)	0.0102 (14)	−0.0017 (16)	0.0066 (16)
C12	0.038 (2)	0.081 (3)	0.138 (4)	−0.0081 (18)	−0.020 (2)	−0.014 (3)
C4	0.0324 (16)	0.055 (2)	0.081 (2)	0.0031 (13)	0.0010 (15)	0.0037 (17)
C13	0.078 (3)	0.088 (4)	0.207 (6)	−0.031 (3)	−0.015 (3)	−0.020 (4)

### Geometric parameters (Å, °)

**Table:**

N2—C8	1.287 (3)	C9—C10	1.511 (4)
N2—C1	1.402 (3)	C9—H9A	0.9700
C6—N1	1.382 (3)	C9—H9B	0.9700
C6—C5	1.389 (4)	C10—C11	1.494 (4)
C6—C1	1.390 (4)	C10—H10A	0.9700
C1—C2	1.394 (4)	C10—H10B	0.9700
C8—C7	1.478 (4)	C11—O2	1.193 (4)
C8—C9	1.495 (4)	C3—C4	1.387 (4)
C5—C4	1.373 (4)	C3—H3	0.9300
C5—H5	0.9300	C12—C13	1.375 (6)
N1—C7	1.349 (3)	C12—H12A	0.9700
N1—H1N	0.96 (6)	C12—H12B	0.9700
O3—C11	1.316 (4)	C4—H4	0.9300
O3—C12	1.453 (4)	C13—H13C	0.9600
C7—O1	1.242 (3)	C13—H13A	0.9600
C2—C3	1.375 (4)	C13—H13B	0.9600
C2—H2	0.9300		
			
C8—N2—C1	118.5 (2)	H9A—C9—H9B	107.6
N1—C6—C5	121.0 (2)	C11—C10—C9	111.3 (2)
N1—C6—C1	118.5 (2)	C11—C10—H10A	109.4
C5—C6—C1	120.5 (3)	C9—C10—H10A	109.4
C6—C1—C2	119.3 (3)	C11—C10—H10B	109.4
C6—C1—N2	121.2 (2)	C9—C10—H10B	109.4
C2—C1—N2	119.5 (2)	H10A—C10—H10B	108.0
N2—C8—C7	123.6 (3)	O2—C11—O3	122.4 (3)
N2—C8—C9	121.0 (2)	O2—C11—C10	125.8 (3)
C7—C8—C9	115.4 (2)	O3—C11—C10	111.8 (3)
C4—C5—C6	119.7 (3)	C2—C3—C4	120.6 (3)
C4—C5—H5	120.2	C2—C3—H3	119.7
C6—C5—H5	120.2	C4—C3—H3	119.7
C7—N1—C6	122.6 (2)	C13—C12—O3	110.1 (4)
C7—N1—H1N	118 (3)	C13—C12—H12A	109.6
C6—N1—H1N	119 (3)	O3—C12—H12A	109.6
C11—O3—C12	115.2 (3)	C13—C12—H12B	109.6
O1—C7—N1	121.8 (2)	O3—C12—H12B	109.6
O1—C7—C8	122.7 (3)	H12A—C12—H12B	108.2
N1—C7—C8	115.5 (2)	C5—C4—C3	120.1 (3)
C3—C2—C1	119.8 (3)	C5—C4—H4	119.9
C3—C2—H2	120.1	C3—C4—H4	119.9
C1—C2—H2	120.1	C12—C13—H13C	109.5
C8—C9—C10	114.4 (2)	C12—C13—H13A	109.5
C8—C9—H9A	108.7	H13C—C13—H13A	109.5
C10—C9—H9A	108.7	C12—C13—H13B	109.5
C8—C9—H9B	108.7	H13C—C13—H13B	109.5
C10—C9—H9B	108.7	H13A—C13—H13B	109.5
			
N1—C6—C1—C2	178.5 (3)	N2—C8—C7—N1	−0.2 (4)
C5—C6—C1—C2	−0.4 (5)	C9—C8—C7—N1	179.5 (3)
N1—C6—C1—N2	−0.8 (4)	C6—C1—C2—C3	0.4 (5)
C5—C6—C1—N2	−179.7 (3)	N2—C1—C2—C3	179.6 (3)
C8—N2—C1—C6	1.3 (4)	N2—C8—C9—C10	0.1 (4)
C8—N2—C1—C2	−177.9 (3)	C7—C8—C9—C10	−179.6 (3)
C1—N2—C8—C7	−0.8 (4)	C8—C9—C10—C11	177.0 (3)
C1—N2—C8—C9	179.5 (3)	C12—O3—C11—O2	1.7 (5)
N1—C6—C5—C4	−178.4 (3)	C12—O3—C11—C10	−179.6 (3)
C1—C6—C5—C4	0.5 (5)	C9—C10—C11—O2	−8.1 (5)
C5—C6—N1—C7	178.6 (3)	C9—C10—C11—O3	173.2 (3)
C1—C6—N1—C7	−0.3 (4)	C1—C2—C3—C4	−0.4 (5)
C6—N1—C7—O1	−177.9 (3)	C11—O3—C12—C13	−172.8 (4)
C6—N1—C7—C8	0.8 (4)	C6—C5—C4—C3	−0.5 (5)
N2—C8—C7—O1	178.5 (3)	C2—C3—C4—C5	0.5 (5)
C9—C8—C7—O1	−1.8 (4)		

### Hydrogen-bond geometry (Å, °)

**Table:**

*D*—H···*A*	*D*—H	H···*A*	*D*···*A*	*D*—H···*A*
N1—H1N···O1^i^	0.96 (6)	1.87 (6)	2.827 (3)	179 (5)
C3—H3···O3^ii^	0.93	2.51	3.426 (4)	170

Symmetry codes: (i) −*x*+1, −*y*+1, −*z*; (ii) −*x*+1, −*y*+2, −*z*.

## References

[bb1] Benbow, J. W., Chu-Moyer, Y. M. & Kung, D. W. (2007). Patent No. US 7 202 245 B2,

[bb2] Bruker (2007). *SADABS*, *APEX2* and *SAINT* Bruker AXS Inc., Madison, Wisconsin, USA.

[bb3] Cai, S. X., Huang, J. C., Espitia, S. A., Tran, M., Ilyin, V. I., Hawkinson, J. E., Woodward, R. M., Weber, E. & Keana, F. M. (1997). *J Med. Chem.* **40**, 3679–3686. 10.1021/jm970396y 9357535

[bb4] Farrugia, L. J. (1997). *J. Appl. Cryst.* **30**, 565.

[bb5] Farrugia, L. J. (1999). *J. Appl. Cryst.* **32**, 837–838.

[bb6] Sarges, R., Howard, H. R., Browne, R. G., Lebel, L. A., Seymour, P. A. & Koe, B. K. (1990). *J. Med. Chem.* **33**, 2240–2254. 10.1021/jm00170a031 2374150

[bb7] Sheldrick, G. M. (2008). *Acta Cryst.* A**64**, 112–122. 10.1107/S0108767307043930 18156677

[bb8] Smits, R. A., Lim, H. D., Hanzer, A., Zuiderveld, O. P., Guaita, E., Adami, M., Coruzzi, G., Leurs, R. & de Esch, T. J. P. (2008). *J. Med. Chem.* **51**, 2457–2467. 10.1021/jm7014217 18357976

[bb9] Sona, P., Carta, A. Loriga, M., Zanetti, S., & Sechi, L. (1998). *Farmaco*, **53**, 455–461. 10.1016/s0014-827x(98)00044-5 9836457

[bb10] Spek, A. L. (2009). *Acta Cryst.* D**65**, 148–155. 10.1107/S090744490804362X PMC2631630 19171970

[bb11] Tandon, V. K., Yadav, D. B., Maurya, H. K., Chaturvedi, A. K. & Shukla, P. K. (2006). *Bioorg. Med. Chem. Lett.* **14**, 6120–6126. 10.1016/j.bmc.2006.04.029 16806945

[bb12] Taylor, E. C., McKillop, A. & Ross, R. E. (1965). *J. Am. Chem. Soc.* **87**, 1990–1995.

[bb13] Toshima, K., Kimura, T., Takano, O. T., Shima, Y., Umerzawa, K. & Matsumura, S. (2003). *Tetrahedron*, **59**, 7057–7067.

